# Narrative Review and Clinical Recommendations for Sportsman’s Hernia and Athletic Pubalgia Based on 30 Years of Expert Experience

**DOI:** 10.3389/jaws.2025.15394

**Published:** 2026-01-12

**Authors:** Moshe Dudai, Hannu Paajanen

**Affiliations:** 1 Hernia Excellence, Tel Aviv, Israel; 2 Department of Gastrointestinal Surgery, University of Eastern Finland, Kuopio, Finland

**Keywords:** sportsman’s hernia, athletic pubalgia, laparoscopic treatment, TEP repair, groin nerve entrapment

## Abstract

**Purpose:**

Sportsman’s Hernia and Athletic Pubalgia (SH/AP) typically develop as a result of muscle imbalance and continuous sports-related microtrauma to the groin area. The injury progresses through two phases: initially, SH is localized in the groin soft tissues, while in the advanced stage, AP extends the injury to the pubic bone. Despite increasing clinical recognition of SH/AP, high-quality, large-scale studies remain limited. As a result, extensive clinical experience may help inform understanding and managing the different phases of the injury. This narrative review aims to stream the authors’ expertise—based on approximately 30 years of hands-on experience in diagnosing, treating, and endoscopically managing SH/AP—into a scientific literature and real-world clinical practice.

**Methods:**

An extensive literature review was conducted to present the current knowledge of diagnostic tests, imaging, endoscopic surgery and rehabilitation treatment of SH/AP. Where appropriate, clinical observations drawn from over three decades of surgical experience with SH/AP patients are used to contextualize the evidence.

**Results:**

MRI is a primary imaging tool for suspected SH/AP, particularly in advanced cases or when nerve involvement is suspected but multiple imaging findings may also be present in asymptomatic athletes. Clinical history and physical examination remain crucial in making the diagnosis of SH/AP, guiding imaging decisions as auxiliary test. Surgical treatment of SH/AP is may be indicated after 2–3 months of failed conservative treatment. The totally extraperitoneal (TEP) without or with release of inguinal ligament (TEP-RRT) approaches have been described in the literature with favorable outcomes. Postoperative rehabilitation plays a critical role in functional recovery.

**Conclusion:**

Physical examination and endoscopic surgery remain most effective in SH/AP according literature and authors experience, followed by dedicated controlled athletics muscles rehabilitation program.

## Introduction

The Sportsman’s Hernia and Athletic Pubalgia (SH/AP) was first described as an entity in late 1980s [1]. It is highly complex and develops usually as a result of sports-related continuous microtrauma in the groin area, which progresses slowly and spreads to adjacent regions as the athlete continues their activity [2]. SH/AP consists of two phases of the injury: first SH is localized in the groin and second AP spreading on the injury to the pubic bone [3]. Due to its atypical nature, it took the surgeons many years to understand this developmental process [4, 5].

The complexity of the injury has led to varying opinions over the years, making it difficult to study the condition, diagnose it accurately, and develop appropriate treatment approaches. Effective diagnosis and management of SH/AP requires extensive, long-term experience to achieve a high level of understanding and the ability to perform optimal surgical treatment [6, 7]. Additionally, rehabilitation physiotherapy must be integrated at different stages of recovery [8].

Only few high-quality medical literature based on large-scale patient data, particularly those undergoing surgical intervention, have been accumulated [9]. It has not been possible to publish many guidelines or meta-analysis based on systematically reviewed scientific literature and evidenced based data that meets high research standards. This lack of standardized data has led to inconsistent diagnostic criteria and varied treatment approaches for groin pain in athletes. To address this issue, initiatives like the Doha Agreement have played a pivotal role in establishing a unified terminology and a structured diagnostic framework for groin injuries [6, 7]. Despite these advancements, there remains a critical need for further studies to develop new treatment frameworks.

The limited availability of large-scale, high-quality studies, the value of extensive clinical experience becomes even more significant. Drawing on approximately 30 years of hands-on expertise in diagnosing, treating, and surgically managing a wide range of athletes suffering from SH/AP, the authors aim to bridge the gap between empirical research and real-world practice. Alongside the relevant knowledge available in the medical literature, we aim to provide well-founded literature review and recommendations based on our extensive experience for the accurate diagnosis and optimal treatment of athletes suffering from SH/AP.

### Pathophysiology of SH/AP

SH/AP is almost always chronic microtrauma overuse injury affecting the groin region, primarily seen in athletes engaged in high intensity contact sports that involve rapid directional changes, sudden acceleration, and repetitive twisting motions [2]. SH/AP may also be diagnosed after repetitive physical work or after pregnancy in non-athletes [10, 11]. Unlike traditional inguinal hernias, SH/AP does not involve a visible bulge but rather a progressive weakening of the posterior wall (PW) of inguinal canal and other musculotendinous structures surrounding the pubic symphysis, leading to chronic pain and functional impairment [12].

Key findings of SH/AP include accumulation of microtrauma when high-impact activities generate repetitive shear forces that cause tearing of the abdominal muscle insertions, particularly at the rectus abdominis, obliques and adductor longus attachments and inflammatory response when the persistence of microtears leads to inflammation, edema, nerve entrapment and irritation behind the inguinal ligament, exacerbating pain and dysfunction [13–15]. These result in tissue degeneration when chronic mechanical loading results in degenerative changes in the PW of inguinal canal and tendinopathies, increasing the risk of tendon ruptures and fibrosis [15, 16].

**FIGURE 1:**
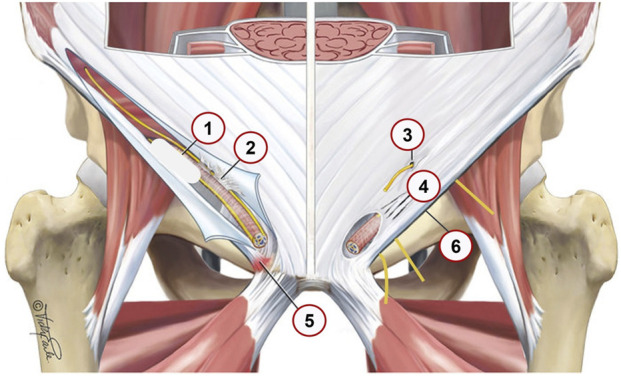
Anatomical locations 1–6 and pathological findings in the inguinal region that contribute to SH grade 1–3. Adapted from Vuckovic; et al. [19]; *BMJ Open Sport Exerc Med*; licensed under CC BY-NC 4.0. Modifications were made. (1) posterior wall bulge–resulting from posterior wall disruption (2) Ilioinguinal nerve adhesions, Fibrotic tissue binding the ilioinguinal nerve; potentially leading to nerve irritation or chronic pain. (3) Ilioinguinal or iliohypogastric nerve entrapment, Compression or entrapment of these nerves; which can result in neuropathic pain. (4) Tears in the external oblique aponeurosis, Disruptions in the aponeurosis that may weaken the abdominal wall and contribute to hernia formation. (5) Enthesopathy at inguinal ligament insertion, Degenerative changes or inflammation at the ligament’s attachment site; potentially causing localized pain. (6) Nerves entrapment behind the inflamed edematous inguinal ligament, Genital and Femoral branch of the Genitofemoral nerve and Lateral Femoral Cutaneous Nerve nerves; it is exacerbating groin pain.

SH/AP involve also progressive structural damage to the transversalis fascia and conjoint tendon leading to PW bulging and theoretically, increased tension in the inguinal canal [3, 12]. Repetitive loading causes stress-related changes, including symphyses disc herniation, bone marrow edema and stress fractures, contributing to chronic pain [17]. The loss of structural integrity in the pelvic stabilizers leads to instability and altered force transmission between the lower extremities and the core [18]. Common aponeurosis of adductors and rectus abdominis muscles may be injured causing chronic pain (Figure 1, adapted from Vuckovic, et al. [19]). Various structural abnormalities in the inguinal region, including nerve entrapments and aponeurotic tears, can contribute to chronic groin pain and should be carefully assessed in clinical evaluations.

**FIGURE 2:**
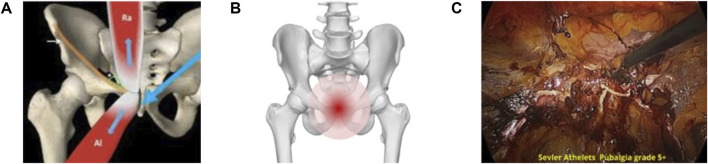
Asymmetric load transfer to pubic symphysis and related bone stress injury in severe AP grade 4–5 **(A)** The biomechanical imbalance between the rectus abdominis and the adductor longus muscles; causing asymmetric force transmission through the pubic symphysis. Adapted from Corazza; et al. [24]; Athletic pubalgia: a detailed didactic approach; ECR 2015; Poster C-2375. Licensed under an open license. **(B)** An anatomical visualization of the pubic bone; emphasizing the stress-prone pain region in cases of severe AP. Adapted from DBCLS (2015); Wikimedia Commons; licensed under CC BY-SA 2.1 JP. Modifications were made. **(C)** An endoscopic intraoperative view of a high-grade AP case; highlighting severe tissue damage with neovascularization and wiled nerves developed with pubic symphysis pathology and disc posterior herniation region.

Biomechanical imbalances play a crucial role in the progression of SH/AP by compromising pelvic stability and altering force distribution. Deficient activation of the transversus abdominis and oblique muscles increases strain on the pubic symphysis and bone [3, 18]. Weakness in pelvic stabilizers allows excessive movement at the symphysis pubic, contributing to recurrent pain and dysfunction and pubic bone stress injury [20–22]. An imbalance between the adductor complex and rectus abdominis leads to excessive force concentration at the pubic symphysis and pubic bone, accelerating stress injury at the pubic bone and symphysis [23]. This process is illustrated in Figure 2A (adapted from Corazza, et al. [24]), Figure 2B (adapted from [25], and Figure 2C).

Entrapped inguinal nerves transmit chronic pain and play a significant role in SH/AP pathophysiology [13, 26]. Edema and inflammation of the inguinal ligament can irritate nerves coursing behind it—particularly the genital (GN) and femoral (FN) branches of the genitofemoral nerve and the lateral femoral cutaneous nerve (LCN)—producing pain and paresthesia in the groin, scrotum, and upper anterolateral thigh [27, 28]. The ilioinguinal (IIN) and iliohypogastric (IHN) nerves traverse between the abdominal wall muscles and are prone to entrapment within these planes. High-resolution ultrasound delineates their course and supports targeted infiltration when indicated [29, 30]. With persistent microtrauma, fibrosis about the inguinal canal and pubic region further narrows these spaces and perpetuates neural irritation [13, 26]. Clinically, pain may radiate along the distribution of the genitofemoral nerve to the upper inner thigh, along the LCN to the lateral thigh, and along the obturator nerve toward the pelvic floor and medial thigh. Symptoms commonly worsen with Valsalva maneuvers or hip extension [27, 28, 31].

SH/AP must be distinguished from traditional inguinal hernias, as misdiagnosis leads to ineffective treatments and prolonged recovery. The floor of inguinal canal may be palpable and painful when examining using index finger and increasing intra-abdominal pressure (Valsalva effect). The absence of a visible bulge differentiates SH from classic hernias, where the herniation of abdominal contents is evident [12]. It should be differentiated from the width PW bulging during strain of the inguinal wall in case of SH [3, 20]. While inguinal hernias can be visualized via ultrasound (US) [32], SH/AP required magnetic resonance Imaging (MRI) and dynamic imaging in early case range 1–3 [33].

The natural progression of SH/AP injury from chronic continues microtrauma to the injury of tendons and muscles and then to the pubic bone and symphysis will happen as the athlete continues his sport activity [34].

The severity of the injury can be graded between 1 to 5 [35, 36]:

- Grade 1: Minor groin ligament or muscle injury [14].
- Grade 2: Involvement of the adductor and rectus abdominis muscles [37, 38].
- Grade 3: Inflammation of prepubic aponeurosis. PW bulging [39, 40].
- Grade 4: Symphysis joint damage with disc herniation [41, 42].
- Grade 5: Pubic bone injury with bone marrow edema and stress fractures [43–45].

### Symptoms and Physical Examination

Given the absence of a visible hernia, SH/AP is often misdiagnosed or confused with other groin pathologies [10]. This section critically examines the key symptoms and physical examination findings necessary for an accurate diagnosis, providing a clear differentiation between SH/AP and other groin-related conditions. SH/AP typically manifests as chronic obscure groin pain that worsens with sports activities, particularly those requiring cutting, sprinting, or twisting motions [10, 20]. The pain is typically unilateral initially but can become bilateral and central lower abdomen as the injury progresses. It does not correlate with a visible bulge, distinguishing it from inguinal hernias [3]. Pain may initially be localized to the groin or proximal adductors (Grades 1–3) as well, but without intervention, pain can spread to the pubic symphysis, lower abdomen, and upper inner thigh [18]. In advanced cases (Grades 4–5), pain may persist even at rest and during sleeping, significantly impairing athletic performance and daily activities [46]. Sharp, burning neuropathic type pain during activity, followed by a dull ache post-exercise is often observed [47]. Pain is exacerbated by core muscle contractions, such as coughing, sneezing, or sudden changes in intra-abdominal pressure [2, 20]. Adductors squeeze usually provokes groin pain in SH/AP [48].

Tenderness along the pubic symphysis and adductor longus insertion suggest pubic symphysis disc herniation or damage and underlying muscular and tendinous involvement [3, 15]. Pubic bone tenderness when compressed indicates stress injury and bone marrow edema with incipient stress fractures [3, 34]. Pain above the pubic bone suggests tendon strain of the pyramidalis and rectus muscles [3, 23]. Pain on direct palpation with index finger during Valsalva’s over the PW indicates weakness of the transversalis fascia and nerve irritation [3,49]. Pain exacerbation with resisted hip adduction or sit-ups highlights instability of the core musculature [18].

Dynamic Examination with Valsalva maneuver may reveal PW weakness of the inguinal canal with PW bulging and pain intensifies upon straining [3, 50]. Single-leg stance test may reveal instability and pain in the pelvic region and suggest pubic symphysis involvement [51]. Resisted sit-up test can elicit pain along the rectus abdominis insertion and pubic symphysis, differentiating SH from adductor strains [52, 53]. Positive FADIR and FABER (flexion, adduction, internal/external rotation of hip) pain suggest rather hip joint-related pain than SH/AP. All other diseases causing lower abdominal or groin pain, have to be excluded (i.e., urological, gynecological, alimentary tract etc) [52].

Isolated adductor strain presents with localized pain and weakness at adductor origin, primarily affecting the adductor tendons [54]. Adductor strain can be part of the SH/AP injury, but can be also an isolated injury. Contrary to adductor strain, SH/AP involves deep-seated pain with nerve-related symptoms, often including pubic symphysis instability [2, 3].

To sum up, early and accurate diagnosis of SH/AP is crucial, as unjustified surgical intervention can cause unnecessary trauma and lead to complications without providing therapeutic benefit. Conversely, delayed more than 3 months or inadequate treatment can result in progressive worsening of symptoms and damage to the pubic bone and symphysis grade 4–5 injury, leading to chronic dysfunction and long-term disability. Failure to identify and treat nerve involvement can result in misdiagnosis and ineffective treatment.

### Imaging Techniques for Diagnosis

Imaging techniques are auxiliary tests in confirming diagnoses, differentiating SH/AP from other musculoskeletal and hip joint conditions, and assessing severity [34, 55]. This section evaluates the effectiveness of US, MRI, computed tomography (CT), and diagnostic laparoscopy during surgery of SH/AP.

US remains a widely used, cost-effective first-line imaging technique for assessing inguinal hernias, but for SH/AP more specific experts’ radiology is required. US utility is also limited due to operator dependency and variable sensitivity. Dynamic US is particularly effective for detecting PW bulging, differentiating SH from inguinal hernia [34]. The Valsalva maneuver, increasing intra-abdominal pressure, enhances visualization of soft tissue disruptions that may not be evident at rest. Adductor strain/tears and prepubic edema can also be demonstrated by the US [18].

MRI is widely considered the gold standard for detecting grade 4–5 injury: symphysis disc herniation, bone marrow edema, stress fractures and muscle or tendon injuries in SH/AP [34]. MRI is also excellent imaging modality for lateral hip-related groin pain [55]. Not enabled by US, MRI is excellent for detecting bone marrow edema, stress fractures, pubic symphysis inflammation, disc herniation, adductor tendon and rectus insertion pathology, helping establish the diagnosis of advanced case grade 4–5 AP and differentiate from isolated adductor tendinopathy [17, 22, 34, 56].

MRI is excellent to rule out other groin and hip injuries often observed in athletes (bursitis, lipomas, muscle sprains etc.) [40]. CT is less frequently used but may be helpful in specific cases when detecting pubic symphysis abnormalities and chronic stress-related changes, hip joint and posterior inguinal wall deficiency [55, 57, 58]. Due to high radiation exposure, CT is not the preferred choice for routine sports injury evaluation [59].

While pre-operative imaging relies on targeted US and MRI to support the clinical diagnosis and assess severity of SH/AP, definitive confirmation and grading of severity of SH/AP is achieved intra-operatively. The final diagnosis and staging are established at the beginning of surgery, based on direct visualization of the posterior wall, pubic bone, and surrounding structures [3, 18]. In a TAPP approach this corresponds to a brief diagnostic laparoscopy with transabdominal inspection [60]. In TEP/TEP-RRT, the extraperitoneal dissection itself provides panoramic exposure of the posterior wall and pubic bone, enabling precise assessment of the extent of injury [3, 49]. This intra-operative confirmation is not used as a stand-alone pre-operative diagnostic test, but rather as part of the indicated surgical treatment after failure of conservative management [3].

It should be noted that many imaging findings can also be observed in asymptomatic volunteers or athletes after heavy training, for example, transversalis fascia bulging, bone marrow edema and adductor tendon irritation [22, 34]. On the other hand, SH/AP may not always be detected on MRI or US, as muscle disruptions are often microscopic and not edematous in grade 1–3. In fact, pelvic MRI can also be normal in grade IV-V injuries [18, 61].

To sum up, MRI should be the primary imaging tool for suspected SH/AP, particularly in advanced cases grade 4–5 or when nerve involvement is suspected. Multiple imaging findings may be present also in asymptomatic athletes. US is valuable as a first-line diagnostic tool in grade 1–3 injuries, but should be interpreted with caution due to operator dependency. Despite imaging technological advances, clinical history and physical examination remain crucial in making the diagnosis, guiding imaging decisions as auxiliary tests.

### Indications for Conservative Treatment Versus Surgery

Conservative treatment serves as the first-line approach, particularly for mild to moderate cases (grades I-III), while surgical intervention is considered for refractory cases, when significant painful structural damage is present. Accurate diagnosis and evidence-based decision-making are essential to tailoring the optimal treatment strategy for each patient [62].

Structured physiotherapy remains the first-line treatment, with a particular emphasis on core stability and adductor muscle strengthening. Holmich et al. demonstrated that active physical training is significantly more effective than passive treatment methods in alleviating chronic groin pain and promoting a successful return to sports [63]. Conservative treatment is recommended for acute or subacute cases with symptoms persisting less than 6–12 weeks, provided no severe muscle or tendon damage is evident [37] and athletes experiencing mild-to-moderate symptoms, where pain is activity-dependent and does not persist at rest [64].

Physiotherapy should include core strengthening exercises as active weight burning exercises to restore pelvic stability and prevent further injury [65], neuromuscular training focusing on adductors, oblique and rectus abdominis muscle co-ordination to improve functional biomechanics [18] and the Copenhagen Adductor exercise program [66].

Pain management also include non-steroidal anti-Inflammatory drugs (NSAIDs) and corticosteroid injections for very short-term severe inflammation control, cryotherapy and US therapy to reduce localized inflammation in acute phase [3, 67]. Athletes are advised to limit high-impact activities while incorporating low-load exercises to maintain conditioning [2].

Expected outcomes in conservative therapy should improve symptoms typically within 4–6 weeks with structured rehabilitation [68]. Return to sport is expected within 8–12 weeks if functional gains are observed [18]. After 6 weeks trail of conservative structure rehabilitation, re-evaluation is performed by the surgeon. If symptoms persist beyond 2–3 months, advanced imaging and reconsideration of surgical intervention are necessary [3]. Top-level professional athletes should be operated already after 2 months of conservative treatment [20].

Surgical intervention is recommended when conservative management fails or when imaging reveals painful structural pathology (grade 4–5) requiring repair. Also, recurrent or worsening pain, limiting an athlete’s ability to train or compete at previous level should be referred to surgical treatment [69]. Significant tendons or muscle tears, particularly affecting the adductor longus or rectus abdominis suggest also surgical repair [2]. Also, nerve involvement, such as GF or other inguinal nerve entrapment, confirmed through imaging and clinical findings is an indication for surgery [4].

Historically identified in between 1980–90, SH surgery has spurred the development of various surgical interventions, from conventional open to minimally invasive techniques (both open and endoscopic) [1]. Transabdominal laparoscopic repair (TAPP) provides more limited exposure of the extraperitoneal groin with good visualization of intra-abdominal structures and carries a higher risk of nerve irritation or other intra-abdominal complication [18]. Certain endoscopic and laparoscopic approaches specifically target the dual pathophysiological components of the pain: nerve entrapment due to inflammatory pressure (TEP release and reinforce technique [TEP-RRT]) [70–72] and structural painful weakness in the groin wall (TAPP Lloyd Release) [73].

To sum up, surgical treatment is needed in recurrent or worsening groin pain preventing athletic performance, pain even on regular daily activity, associated with complete tendon or muscle tears (e.g., adductor longus, rectus abdominis) or entrapment of inguinal nerves. Surgery should be considered after 2–3 months of failed conservative treatment when imaging reveals grade 4–5 groin injury, including painful (pain scores >7, range 0–10) PW deficiency, symphysis pathology, disc herniation, bone marrow edema or the pyramidalis–anterior pubic ligament–adductor longus complex (PLAC).

### Surgical Approaches for the Treatment of SH/AP

Over the years, several surgical approaches have been developed for the treatment of SH/AP, aiming to address the wide spectrum of underlying pathophysiological factors—including PW deficiency of the inguinal canal, pubic bone pathology, and chronic nerve entrapment.

Open surgical techniques are generally suitable for moderate cases (grades 1–3), particularly when conservative treatments fail. In such cases, suture-based reinforcement of the PW is commonly performed without mesh placement, as anterior mesh implantation may cause irritation in active athletes [74]. In some cases, such as recurrent or anatomically complex presentations, open techniques are considered when laparoscopy is contraindicated [49]. However, these approaches do not comprehensively address all components of the injury, including biomechanical deficiency, osseous pathology, and nerve involvement [75].

A prominent open technique is the minimal repair developed by Muschaweck, which is performed under local anesthesia, avoids mesh usage, and targets localized PW weakness with concurrent pressure on the genital branch of the genitofemoral nerve [74, 76]. Notably, Muschaweck provided histological evidence of chronic nerve injury: in 100% of cases where neurectomy was performed, perineural fibrosis was observed—indicating chronic inflammatory processes likely contributing to persistent pain. Surgical excision of the fibrotic nerve segment resulted in significant clinical pain relief, supporting the hypothesis that nerve entrapment is a primary source of symptoms in a subset of patients. Nevertheless, this approach is not suitable for advanced cases (grades 4–5) involving extensive pubic pathology [74, 76, 77].

A laparoscopic alternative is the TAPP approach, in which repair is performed through the peritoneal cavity, and is suitable for patients with grades 4–5 SH/AP [78]. A specialized variant, known as the Lloyd release, involves laparoscopic tenotomy of the inguinal ligament near its insertion at the pubic tubercle, followed by mesh reinforcement [73]. Histological studies have demonstrated degenerative changes in the ligament, supporting its role as a pain generator. This release aims to reduce tension and chronic inflammation in the inguinal ligament, similar to procedures used in orthopedics (e.g., tennis elbow), in which ligament release reduces stress and improves symptoms. Mesh placement after the release provides structural support [79]. Clinical outcomes demonstrated pain improvement, with over 90% of athletes returning to activity within 4–6 weeks SH/AP [73, 80].

Another minimally invasive standard approach is TEP repair, as described in the work of Paajanen et al., who reported that over 90% of patients with grades 4–5 return to full activity without pain [20, 81, 82]. TEP provides a comprehensive repair by reinforcing the PW, treating pubic symphysis and bone pathology, and restoration of structural integrity through the placement of a midweight (40–60 g/m^2^) polypropylene mesh on PW and pubic. Furthermore, the use of biological glue instead of staples or sutures helps reduce postoperative neuralgia. The procedure is performed extraperitoneally, minimizing the risk of intra-abdominal complications and enable widely exposure the entire bilateral groins and the pubic bone [20, 81, 82].

**FIGURE 3:**
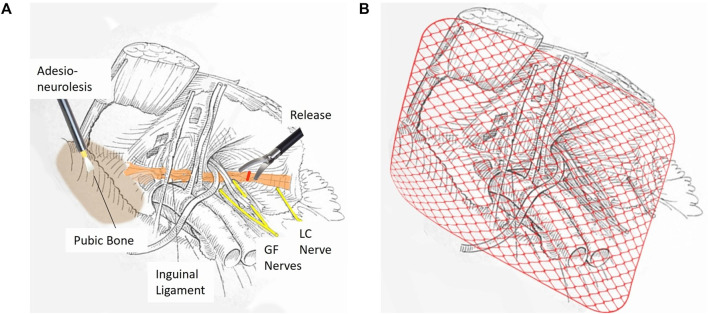
Two-Stage TEP-RRT Endoscopic Surgery for Sports Hernia Repair. **(A)** First Stage: Release. This stage focuses on the separation of extensive adhesions and meticulous cleaning around the pubic bone. An adhesio-neurolysis is performed to release entrapped nerves; followed by decompression of the ilioinguinal and genitofemoral (GF) nerves; which may be compressed by the IL. A precise release of the ligament is executed to alleviate nerve entrapment and reduce chronic groin pain. **(B)** Second Stage: Reinforce. In the final stage; the repaired area is reinforced by placing a mediumweight surgical mesh that covers the entire inguinal region. The mesh is fixed using absorbable biological glue; ensuring stable coverage while minimizing the risk of chronic pain and foreign body reactions.

Dudai has described the development and refinement of TEP-RRT—a bilateral endoscopic approach that builds upon the TEP framework and has evolved over three decades of clinical practice. It directly targets the dual pathophysiology of SH/AP: biomechanical instability and chronic nerve entrapment [70–72]. The procedure is divided into two distinct phases: release and reinforce (1) Release–Adhesiolysis and excision of scar tissue and lipomas, debridement of the pubic symphysis and bone, and inguinal ligament division to decompress the tree entrapped nerves below (Figure 3A). (2) Reinforce–PW and pubic bone by placement of a 13 × 16 cm midweight mesh using biological glue (no sutures or staples) to avoid nerve irritation and enhance stability (Figure 3B) [70–72].

The mid-ligament division of the inguinal ligament is performed at the point of nerve passage (GN, FN, LCN), in contrast to the medial release in the Lloyd technique, where the nerves are not typically entrapped. The proposed mechanism suggests that repeated microtrauma induces thickening and fibrosis of the inguinal ligament, resulting in a loss of elasticity and the development of pathological tension on adjacent neural structures. Thus, this targeted division relieves direct pressure from a fibrotic, thickened ligament on the nerves without damaging them, thereby allowing for their recovery and restoration of normal function—analogous to the principle applied in carpal tunnel release surgery, where decompression is performed while preserving the integrity of the nerve. In addition, this targeted division also contributes to reduced tension and chronic inflammation within the inguinal ligament itself, similar to the effect achieved by the Lloyd technique [70–72].

Growing clinical, anatomical, and histological evidence underscores the central role of nerve entrapment—often exacerbated by chronic inguinal ligament strain—in the persistence of SH/AP symptoms. One non-surgical intervention targeting this mechanism is Radiofrequency Denervation (RFD), which utilizes high-frequency radio waves to disrupt nociceptive signal transmission in regions affected by chronic inflammation, particularly around the inguinal ligament. This technique provides a conservative treatment option for patients with neuropathic pain. However, it is not suitable for advanced SH/AP cases (grades 4–5), which involve both structural and pubic bone abnormalities and complex neural involvement [83, 84].

Taken together, the integration of inguinal ligament release into SH/AP surgery represents a targeted and pathophysiologically grounded strategy for relieving both inflammatory tense and nerve entrapment. This maneuver serves as a key adjunct to the classical TEP technique and may significantly contribute to improved long-term surgical outcomes.

The effectiveness of TEP-RRT is substantiated by two large-scale studies conducted between 2016 and 2024, involving a total of 461 athletes—most of whom suffered from high-grade (4–5) SH/AP—underscoring the efficacy of TEP-RRT and providing strong evidence for its clinical application [70–72]. The full outcome data from this cohort will be detailed in a forthcoming peer-reviewed publication by Dudai, et al.

Table 1 presents a comparative summary of key outcome measures from two studies evaluating the effectiveness and safety of endoscopic surgical treatment for SH/AP.

**TABLE 1:** Outcomes of TEP-RRT for SH/AP: summary of two studies.

Outcome	Study 1 (prospective; 2017–2018; N = 150)	Study 2 (retrospective; 2016–2024; N = 461)
Nerve entrapment resolution	96% pre-op → 1.3% at 8 weeks post-op	-
Return to sport and remain active	100% returned	98.5% (454/461)
Time to return to sport	Within 5–10 weeks	75% ≤ 8 weeks; 25% ≤ 12 weeks
Recurrence rate	0 cases	0 cases
Complications	1.3% (rectus hematomas) no persistent neuropathy	1% (7/461) (mesh infection; deep vein thrombosis; rectus hematomas; thermal injury)
Additional treatment	-	10% needed additional physio/pharma
Patient satisfaction	-	96% rated outcomes as “very good”

In Study 1: 2017-18 prospective study of 150 patients, 96% of athletes presented with thigh nerve entrapment (TNE) preoperatively, pain dropped to 1.3% 8 weeks after surgery (McNemar’s test, p < 0.0001). All athletes returned to sport within 5–10 weeks, with no reports of chronic pain. The complication rate was low (1.3%), with no cases of persistent neuropathy [72] (Table 1).

In Study 2: 2016-24 retrospective long-term outcome survey conducted via personal telephone interviews of 461 patients. 98.5% of athletes returned to sport and remain active (Table 1). 7 patients had early post operative complications, but 6 of the completed the AMRP and returned to sport activity. 14 patients with recurrent SH/AP operated in other centers underwent revision surgery and all returned to sport activity. The recurrence rate was minimal 0% and the overall patient satisfaction was high (96%) [70] (Table 1).

Challenges and limitations of TEP-RRT include need for specialist training. Surgeons require specific expertise in both hernia and sports injuries to perform the procedure and to follow the athlete. Although TEP-RRT is designed as a nerve-sparing approach, suboptimal technique may lead to postoperative neuralgia or transient sensory disturbances. Careful dissection and anatomical precision are critical to avoid nerve injury during inguinal ligament release. Strict adherence to rehabilitation programs (see later) is essential for successful recovery and preventing recurrence. Despite compelling clinical results, existing studies are limited by lack of a control group. To further validate the superiority of TEP-RRT, prospective randomized controlled trials are needed [70–72].

To sum up, endoscopic (laparoscopic) techniques should be the preferred surgical approach for treating SH/AP in athletes, due to their lower complication rates, faster recovery, and superior functional outcomes compared to open surgery. TEP repair is recommended over TAPP to avoid intra-abdominal entry, thereby minimizing the risk of adhesions, bowel injury, and peritoneal complications. In addition, TEP provides wider surgical exposure than TAPP, facilitating access to the bilateral groins and pubic bone. TEP-RRT should be prioritized in moderate to severe (Grade 4–5) SH/AP cases, as it uniquely addresses all three core pathologies: nerve compression (via inguinal ligament release), PW instability (via mesh reinforcement) and pubic bone pathology (via debridement and stabilization).

### Postoperative Structured Rehabilitation Program

Rehabilitation programs play a central role in the management of SH/AP, both as a conservative treatment approach and following surgical intervention. In the early stages of the condition (grades 1–3), active rehabilitation is commonly employed as a first-line therapy to reduce pain, promote soft tissue healing, improve core and hip muscle stability, and restore athletic function—ideally avoiding surgery. Reflecting this emphasis, most published studies have focused on conservative rehabilitation protocols [8, 85]. When a clear structural defect is identified in the abdominal wall or tendon, or when conservative management fails, surgical intervention becomes necessary. However, surgical success does not rely solely on the operation itself, rather, it is highly dependent on a structured, gradual, and personalized rehabilitation process. In fact, rehabilitation is considered a critical determinant of long-term outcomes. Despite this, most surgical studies tend to focus primarily on the technical aspects of the surgical procedure, often describing the postoperative rehabilitation only briefly—sometimes in vague terms such as “a stepwise return to activity beginning within 2 weeks postoperatively.” The lack of detailed postoperative rehabilitation protocols represents a significant gap in the literature [86].

Nevertheless, there are notable exceptions. For instance, Palumbo, et al. [87] presented a structured, 3-month rehabilitation protocol including core strengthening, adductor muscle training, single-leg stance stability, and patient-specific adaptations based on individual response. The rehabilitation process was continuously monitored by physiotherapists, ensuring personalized progression and pain-free training. Their results indicated complete pain resolution in 32 of 34 athletes who underwent open repair between 2017 and 2019—suggesting high protocol efficacy. However, follow-up was short-to mid-term, and recurrence rates were not reported [87].

Another structured rehabilitation protocol is the athletic muscle rehabilitation program (AMRP), developed based on over 25 years of clinical experience and currently integrated as the standard postoperative rehabilitation following endoscopic TEP-RRT surgery. The program is built on close collaboration between the operating surgeon and physiotherapists. Its unique structure consists of three well-defined phases aligned with the physiological and functional healing process. Table 2 outlines the three-phase of AMRP. Stage 1 (Weeks 1–3) focuses on early mobilization with isometric and weight-bearing exercises to promote core stability and prevent stiffness, with light activity encouraged from week one. Stage 2 (Weeks 4–6) includes progressive strength training targeting the rectus abdominis, adductors, and pelvic stabilizers and advanced coordination. Stage 3 (Weeks 7–12) emphasizes aerobic conditioning and sport-specific drills. Return to sport is based on functional assessment by the surgeon. The AMRP is distinct in that it does not end with return to activity, rather, it includes long-term maintenance strategies to prevent reinjury and preserve functional gains. Recurrence prevention involves warm-up routines, biomechanical correction, and ongoing strength training program even during brake and rest. Recovery duration may vary based on injury severity. Long-term outcomes of the AMRP were evaluated in a study including 461 athletes who underwent TEP-RRT surgery between 2016 and 2024. The results demonstrated that 98.5% of patients returned to full athletic activity, with no reported cases of recurrence. The large sample size and extended follow-up period strengthen the significance of these findings [35, 70].

**TABLE 2:** Stages of the athletic muscle rehabilitation program (AMRP)– structure; goals; and key exercises.

Stage	Time frame within the process	Rehabilitation goals	Key exercises	Additional recommendations
Stage 1: early mobilization and strengthening	Weeks 1–3	Activate core stability; prevent stiffness	Isometric exercises; weight-bearing movements	Avoid high-impact activities to allow tissue healing
Stage 2: progressive strength and functional training	Weeks 4–6	Improve core and pelvic stability; and strengthen specific muscles coordination	Exercises targeting rectus abdominis; hip adductors; and pelvic stabilizers	Incorporate progressive resistance training for neuromuscular control
Stage 3: preparing for return to full activity	Weeks 7–12	Restore full sports functionality	Aerobic training; sport-specific drills	Perform functional assessments before returning to competition

To sum up, surgical success in treating SH/AP relies not only on advanced operative technique, but also on the implementation of a structured, progressive, and individualized rehabilitation program. The AMRP comprehensively addresses this need and stands out as one of the few postoperative rehabilitation protocols that is systematically documented and outcome-based. Its combination of clearly defined phases, ongoing supervision, active surgical involvement, and personalized adaptation positions the AMRP as a leading expert-recommended protocol for postoperative recovery—both for its clinical effectiveness and its role in minimizing recurrence.

### Treatment of Recurrent Injuries

Despite advancements in laparoendoscopic repair and rehabilitation protocols, a subset (<3%) of athletes continues to experience chronic pain and reinjury after endoscopic surgery. The recurrence of SH/AP is often linked to incomplete healing, biomechanical imbalances, or inadequate rehabilitation. Recurrent SH/AP differs from acute cases in that it develops post-treatment due to incomplete tissue recovery [35]. Chronic groin instability and persistent pain are hallmark features, often leading to secondary pubic bone stress injuries (PBSI). Core muscle imbalances and adductor dysfunction contribute to abnormal stress distribution in the groin [35]. Residual nerve entrapment post-surgery is a major contributor to persistent pain. Undiagnosed hip injury may be present or AMRP is not performed in proper way postoperatively [35].

Non-surgical options are emphasized for early stage of recurrent SH/AP and AMRP is as the primary intervention [35]. Pain management should include NSAIDs, corticosteroid injections, cryotherapy and hyperbaric oxygen therapy in high pressure chamber, if this treatment is available [3]. Indications for surgical revision are failure of conservative treatment to return to sport activity. Grades IV-V injuries require revision surgery to reinforce PW deficiency and pubic bone pathology [35]. Persistent nerve entrapment cases necessitate endoscopic nerve release. In failed SH/AP surgery the hip joint-related problems must be excluded and ortopedic consultation is necessary in many cases. Also isolated adductor tendinopathies may need tenotomies [35].

Preferred surgical procedure in recurrent cases is TEP. When reoperating, the original mesh has to be removed from anterior wall [3]. Advanced adhesive mesh fixation techniques reduce chronic pain recurrence. In TEP-RRT, release of the genitofemoral, ilioinguinal, and lateral femoral cutaneous nerves is frequently necessary. Progressive aerobic loading to restore cardiovascular endurance should be encouraged. Dudai reports that out of the 461 patients he operated on, 14 patients underwent revision TEP-RRT following failed SH/AP surgery elsewhere. All of them completed the rehabilitation program within 8 weeks and returned to sports activity [70].

To sum up, the management of recurrent SH/AP injuries requires a comprehensive and individualized approach that addresses the root causes of recurrence—most commonly incomplete AMRP and healing, biomechanical dysfunction, and residual nerve entrapment. Early-stage recurrences should be treated conservatively with a structured AMRP program and targeted pain management. The preferred surgical strategy should combine anatomical restoration with nerve decompression, while minimizing risks of re-injury through advanced fixation techniques and biological adhesives.

### The Recommended Clinical Algorithm for the Diagnosis, Treatment, and Rehabilitation of SH/AP

**FIGURE 4:**
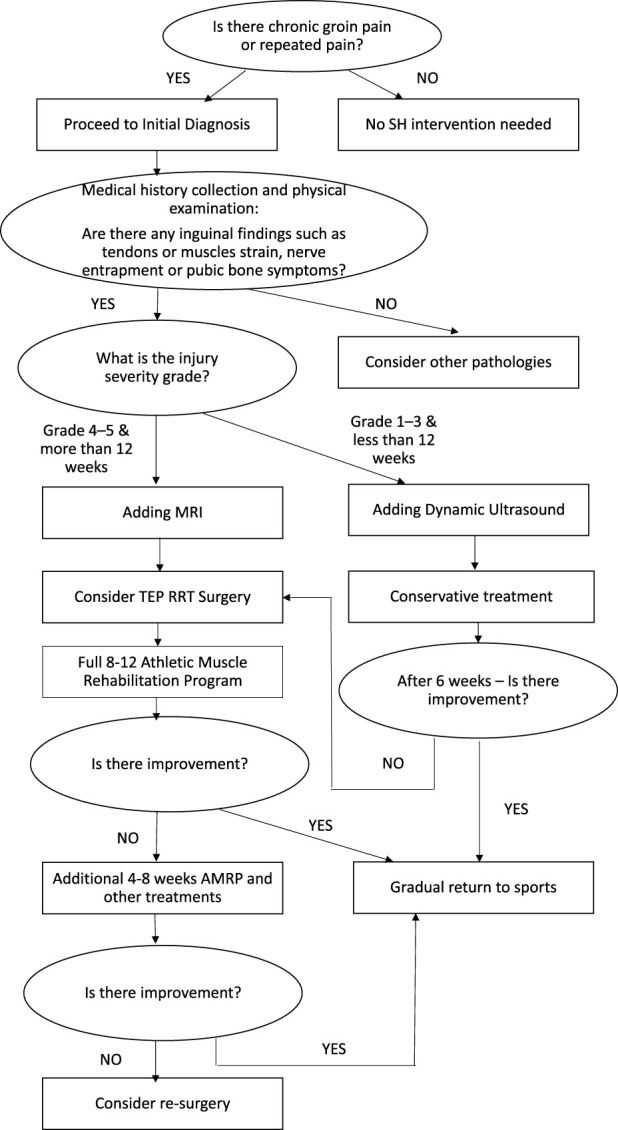
Flow sheet - Diagnosis; Treatment; and Rehabilitation of SH and AP.

Figure 4 presents a flowchart summarizing the diagnostic process, treatment pathways, and rehabilitation strategy for SH and AP.

## Conclusion

SH/AP are progressive injuries that develop due to continue chronic microtrauma long as the athlete continues his activity on the injury, leading to a gradual progression from localized soft tissue injury in early stage 1–3, to the most severe pubic bone pathology grade 4–5.

Accurate diagnosis of SH/AP relies on history and primarily on physical examinations but can be supported by advanced imaging techniques. MRI remains the preferred choice, particularly when symphysis and pubic bone involvement is suspected. MRI also excludes most accurately other injuries in the groin area (hip joint and muscles/tendons). Positive imaging findings in the groin area are found in asymptomatic athletes as well.

TEP or TEP-RRT has emerged as the preferred surgical approach for SH/AP due to its low recurrence rates, rapid recovery, and superior pain resolution. TEP-RRT uniquely addresses all three major pathophysiological components of SH/AP: (1) Nerve decompression (2) Biomechanical stabilization (3) Correction of pubic bone pathology. TEP-RRT is recommended for the preferred choice for athletes and physically active individuals because of its significant results of 98.5% return to sport activity.

Rehabilitation is mandatory and plays a pivotal role in long-term recovery. Adoption of TEP or TEP-RRT as the preferred surgical method, combined with AMRP for post-surgical rehabilitation, can significantly improve in recurrence prevention and return-to-sport rates.
